# Characterizing the transplanar and in-plane water transport of textiles with gravimetric and image analysis technique: Spontaneous Uptake Water Transport Tester

**DOI:** 10.1038/srep09689

**Published:** 2015-04-15

**Authors:** K. P. M. Tang, Y. S. Wu, K. H. Chau, C. W. Kan, J. T. Fan

**Affiliations:** 1 Institute of Textiles and Clothing, the Hong Kong Polytechnic University, Hung Hom, Hong Kong; 2 Department of Fiber Science and Apparel Design, College of Human Ecology, Cornell University, Ithaca, 14853, NY, United States

## Abstract

Water absorption and transport property of textiles is important since it affects wear comfort, efficiency of treatment and functionality of product. This paper introduces an accurate and reliable measurement tester, which is based on gravimetric and image analysis technique, for characterising the transplanar and in-plane wicking property of fabrics. The uniqueness of this instrument is that it is able to directly measure the water absorption amount in real-time, monitor the direction of water transport and estimate the amount of water left on skin when sweating. Throughout the experiment, water supply is continuous which simulates profuse sweating. Testing automation could even minimise variation caused by subjective manipulation, thus enhancing testing accuracy. This instrument is versatile in terms of the fabrics could be tested. A series of shirting fabrics made by different fabric structure and yarn were investigated and the results show that the proposed method has high sensitivity in differentiating fabrics with varying geometrical differences. Fabrics with known hydrophobicity were additionally tested to examine the sensitivity of the instrument. This instrument also demonstrates the flexibility to test on high performance moisture management fabrics and these fabrics were found to have excellent transplanar and in-plane wicking properties.

Moisture in clothing has been widely acknowledged as one of the fundamental factors causing discomfort during wear^1^,^2^. Fukazawa and Havenith^3^ and Galbraith et al.^4^ found that the major factor causing discomfort is the un-evaporated sweat remained on the skin surface. Despite clothing, the liquid absorption and transport property is important for the health-care products, such as incontinence pads^5^,^6^ and wound dressing products^7^,^8^. Its wetness is often associated with skin wetness and the increased skin wetness may cause dermatitis^5^,^6^,^9^. Despite the comfort perspective, liquid absorption and transport property is important during the processing stage. Fabrics with hydrophilic nature could ensure uniformity, efficiency and evenness of the treatment. These shed light on the importance of studying the water absorption and transport ability of textiles, and properties such as wicking across (in-plane wicking) and through the plane of the material (transplanar wicking – away from the skin). These two directions of wicking are crucial in facilitating the evaporation of sweat and minimizing the wetness sensation of skin, thus it demonstrates the demand of an effective measurement method.

Numerous water absorption tests are currently available^10^,^11^,^12^,^13^,^14^,^15^ (Details can be found in Supplementary Table S1 online). In Tang et al's review article^16^, these test methods are classified according to the technique adopted, including gravimetric, observation-based, optical, spectroscopic, electrical, pressure-based, magnetic resonance-based and temperature detection methods (Advantages and limitations of these techniques can be found in Supplementary Table S2 online). In brief, the conventional testing methods do not simulate the end-use condition of fabric (do not wet the fabric continuously^17^ or do not deliver water to fabric in proper direction^18^), could only apply to certain types of fabrics^19^,^20^, complicated^21^,^22^,^23^ and obtain inadequate information on water transport property^17^. These methods mainly focus on in-plane wicking and cannot differentiate the direction of water transport, so a measurement method for effective investigation of in-plane and transplanar wicking property of fabric is required. The shortcomings and inefficiencies of these conventional methods associated with the growing demand on clothing comfort pose an insatiable desire on a new test method; hence, Spontaneous Uptake Water Transport Tester (SUWTT) is developed. According to Miller and Tyomkin^24^, the term spontaneous means that ‘the movement of liquid takes place against a zero or negative liquid-head pressure gradient’ which is in contrast with the forced wicking where liquid is forced to pass through a fabric. The main contribution of this study is to develop a versatile and automatic measurement method that could differentiate the direction of water transport accurately, repeatedly, simply and could simulate the end use condition of fabric. In-plane and transplanar wicking property affect the evaporation of water within the fabric and are related to long-term wear comfort. Besides, the water absorption rate can be measured which reflect the situation during the initial period of sweating. Moreover, a novel parameter called ‘amount of water absorbed by the bottom filter paper’ is measured and this could reflect the moistness of our skin when sweated. All these measurements suggest that in-depth information regarding wear comfort can be obtained from this instrument.

The usage of SUWTT is demonstrated by investigating the water absorption performance of the 21 types of fabrics. These fabrics, comprising different fabric construction, yarn type, fibre content and varying concentration of water repellent finish, are categorized into three groups. Details of each group and the specifications of each fabric are summarized in Supplementary Table S3.

## Methods

### Design and configuration of the experimental set up

**Figure 1:**
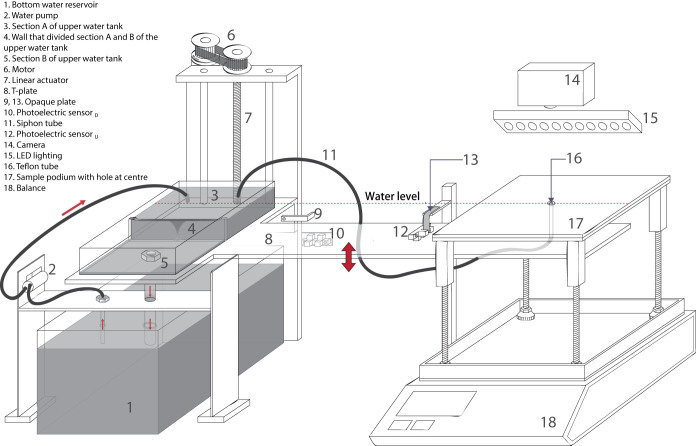
Schematic diagram of the Spontaneous Uptake Water Transport Tester. This diagram shows the hardware configuration of the instrument. It consists of a balance and a camera of which the water absorption property of the sample was characterised by gravimetric and image analysis technique.

Figure 1 illustrates a schematic diagram of the Spontaneous Uptake Water Transport Tester. As shown in Figure 1, gravimetric and image analysis technique are adopted for the measurement. This instrument can directly measure the water absorption amount in the sample podium side (17) in real-time by the balance (18) and capture the wetted pattern of the sample by the attached camera (14). Upper water tank (3, 5) and the Teflon tube of around 2 mm inner diameter (16) were sat on the T-plate (8) connected by a siphon tube (11). Water level in Section A of the upper water tank (3) and the Teflon tube (16) were kept constant throughout the test no matter what level the T-plate was, thus maintaining a constant hydrostatic head and improving the accuracy of the test. The water level in the Telfon tube (16) was about 1 mm above its orifice so that the water droplet could contact with the fabric but not for the tip of the tube. The T-plate was mounted on the linear actuator (7). The movement direction and speed of the T-plate (8) was controlled by the voltage supply to the motor (6). 7.5 V positive voltage contributes to relatively fast downward movement of the T-plate (8) (6.9 mm/min) whilst 3.5 V negative voltage contributes to slow upward movement (2.7 mm/min). The fast downward movement aims to remove the water supply source from the fabric surface sharply after the test.

Before putting the sample onto the sample podium (17), T-plate (8) was lowered so that sample placed onto the sample podium (17) did not contact with the water source. Two photoelectric sensors (10, 12) were used to stop the movement of the T-plate (8). Photoelectric sensor _D_ (10) was attached to the linear actuator (7) and functioned with an opaque plate (9) which helps to determine the end point of downward movement. On the other hand, photoelectric sensor _U_ (12) was mounted on the T-plate (8) and functioned with another opaque plate (13). When the photoelectric sensor _U_ (12) detected the opaque plate (13), the upward movement of the T-plate was stopped. By that time, the Teflon tube (16) has already reached the upper side of the hole (58 mm diameter) located at the centre of the sample podium (17) and the sample started absorbing water. By having one-point water supply, it can ensure that wicking started in the middle part of the fabric and the horizontal wicking performance could be characterised. A camera (14) associated with a set of LED lighting (15) was situated on top of the sample podium (17) to record the wetted pattern of the sample. After supplying water to the sample for a predetermined duration, the T-plate returned to its original position automatically and the test was terminated.

For the water circulation system, similar to the Transplanar Water Transport Tester (TWTT), the water stored in the bottom reservoir (1) was pumped to section A of the upper water tank (3) which helps to maintain a constant water level. The spilled water leaked out across the wall (4) to section B (5) which was equipped with a relatively thick tube for returning the surplus water to the bottom reservoir (1) by gravity. As water was absorbed by or transported through the sample, water was continuously supplied to the sample through siphon action and hence less water returned to the bottom water reservoir (1).

### Arrangement of sample for testing

**Figure 2:**
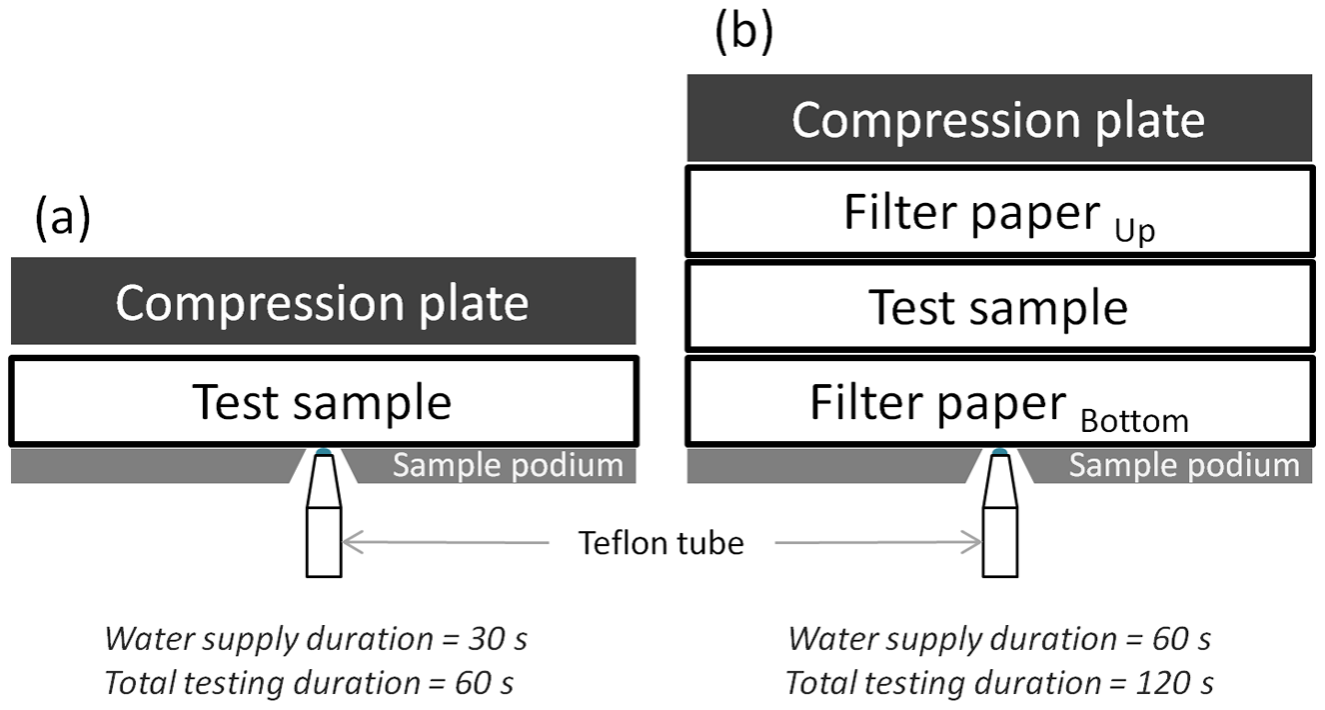
Arrangement of sample for different set up. (a): 1-layer set up of SUWTT with only the sample put onto the sample podium. This setting is for measuring the horizontal wicking property of fabric. (b): 3-layer set up of SUWTT with the sample put in-between two filter papers for characterising the transplanar wicking property of the sample.

These specimens (12 × 12 cm) were conditioned in a standard atmosphere (20 ± 1°C and 65 ± 5% RH) for at least 24 hours prior to testing. This instrument can test either the horizontal wicking property of the sample in a 1-layer set up (as shown in Figure 2(a)) or the transplanar wicking property in a 3-layer set up (as shown in Figure 2(b)). Only the test sample was put onto the sample podium for the measurement of horizontal wicking property whereas filter papers were utilized for measuring the transplanar wicking property. The filter paper put below the test sample is for simulating human skin. The tiny channels in the filter paper, acted as the sweat glands of skin, are for transporting water to the fabric. The mass of water absorbed by the bottom filter paper could be used for estimating skin condition. On top of the test sample was another filter paper, it helps to characterise the amount of water that has passed to the outer surface of the sample. In both of the set up, a compression plate (0.5 g/cm^2^) was put onto the sample to ensure proper contact with the water source and reproducible contact between layers. For the 1-layer test, the water supply duration was set as 30 seconds and the fabric was compressed for additional 30 seconds. In the 3-layer test, the water supply duration was 60 seconds and additional 60 seconds were allowed for layer to layer wicking.

### Measurement parameters

**Figure 3:**
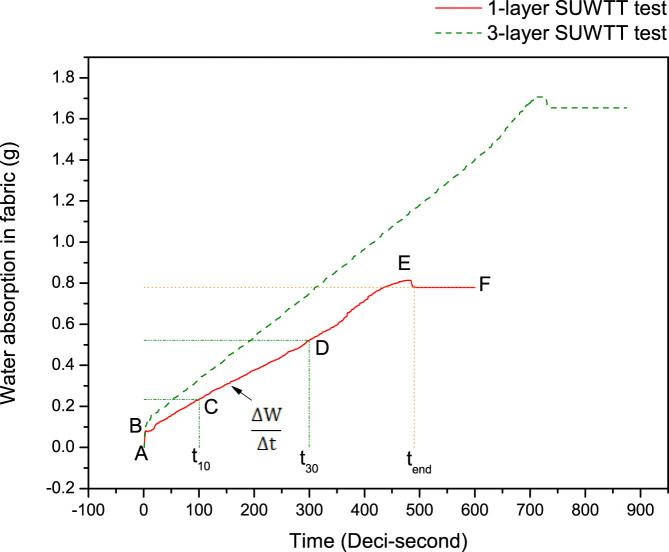
Water absorption curve of a 1-layer and 3-layer SUWTT test for a typical woven fabric. The red solid line indicates water absorption performance of a 1-layer test while the green dotted line reflects the mass of water absorption of a 3-layer test. Point A to B represents the situation once the fabric is in contact with water. During the initial absorption period, the water absorption mass in fabric increases sharply which might attribute to the upward force exerted by the water supply tube. From Point C to D, the rate of increment in the absorption mass is almost constant and this portion is to depict the water absorption rate of fabric. From point D onwards, the water supply tube is moving downward and water supply is discontinued. Still, it can be observed that the absorption amount is increasing. This phenomenon might attribute to the surface tension of water. The hemisphere of water droplet formed on the tip of Teflon tube does not separate from the fabric immediately. Instead, the droplet is stretched and thus water absorption process continues until point E. When the hemisphere of water droplet leaves the fabric completely, its weight exerted on the sample podium part vanishes and so the water absorption amount decreases from point E to point F.

The water absorption amount of the specimen was recorded against time and the water absorption rate was calculated according to the slope between time at 10 second (t_10_) and the set time for water supply (t_set_). In Figure 3, the slope between point C and D implies the water absorption rate of the fabric. The higher the absorption rate, the less likely the water remained between the fabric-skin interface and thus the more comfort it provides.

The wetted area of the test sample was recorded at the end of the experiment. The larger the spreading area, the faster the evaporation rate might be. The spreading area reflects the in-plane wicking property of fabric; however, it is somehow affected by fabric thickness. With everything being equal, thicker fabric tends to have smaller spreading area due to large volume of fabric to be wetted.

Water content focuses on the concentration of water within the sample. It can be calculated with reference to equation (1). This parameter is affected by the geometry of the sample with its wetted area, thickness and porosity being considered. In general, the higher the water content within a fabric, the less comfort it provides.
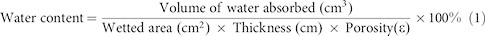


In the 3-layer set up, the amount of water absorbed by the bottom filter paper was additionally recorded. Here, the bottom filter paper acted as a simulated skin layer and it is an indirect estimation of the degree of skin moistness when sweated.

In the 3-layer set up, the testing sample was placed in-between two filter papers. In order to quantify the transplanar water flow, water distribution in the top and bottom filter paper was determined and it is anticipated that this proportion could tell the transplanar wicking ability of the fabric. By dividing the water absorption amount in the top filter paper with the bottom one, an index called ‘Transplanar ratio’ was developed as shown in equation (2). It is believed that the higher the ratio, the farther the water being transported away from the skin surface and the less clammy sensation it provides.
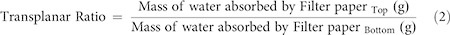


## Results and Discussion

### Typical absorption curve

Figure 3 shows the absorption curve of a typical woven fabric measured by a 1-layer and 3-layer SUWTT test.

### Investigating the usage of SUWTT

**Figure 4:**
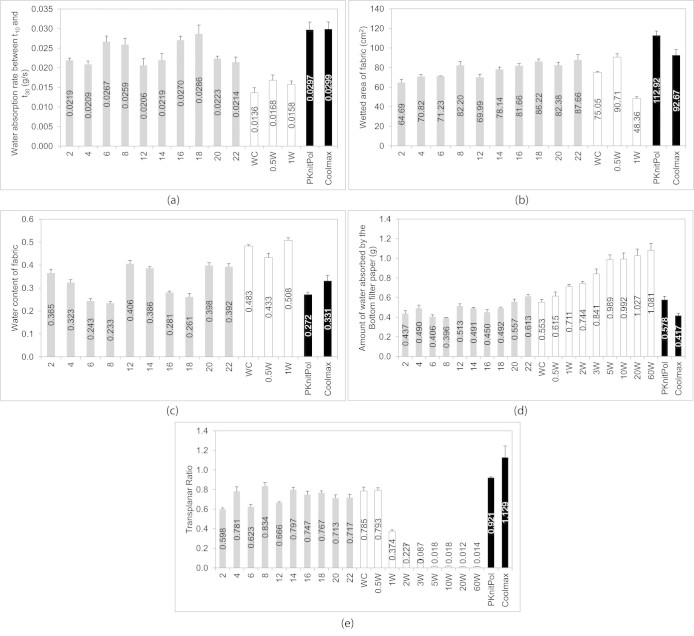
SUWTT results of various fabrics. The bars in grey colour indicate the result of group A's samples while the bars in white and black denotes group B's and group C's samples, respectively. The error bars represent mean ± S.D. of five samples. (a): Water absorption rate of fabric measured in 1-layer SUWTT test. (b): Wetted area of fabric measured in 1-layer SUWTT test. (c): Water content of fabric measured in 1-layer SUWTT test. (d): Amount of water absorbed by the bottom filter paper measured in 3-layer SUWTT test. (e): Transplanar ratio measured in 3-layer SUWTT test.

The measurement results of 1-layer test are illustrated from Figure 4(a) to Figure 4(c) while the results for the 3-layer test are illustrated in Figure 4(d) and Figure 4(e). Between-groups analysis of variance (ANOVA) test was performed to test for the ‘main effect’ for each independent variable on the dependent variable. The significance level of the statistical analysis conducted in this study was set at 0.05. The analysis of this section is divided according to the grouping of the fabrics. Group A aims to examine whether the proposed instrument can differentiate fabrics with different structures and yarn types. Group B, consists of fabrics with known hydrophobicity, aims to examine the sensitivity of the instrument. Group C, comprises of moisture management fabrics, aims to investigate the flexibility of the proposed instrument on testing high performance fabrics.

### Group A – The sensitivity in differentiating fabrics with different geometrical properties

**Figure 5:**
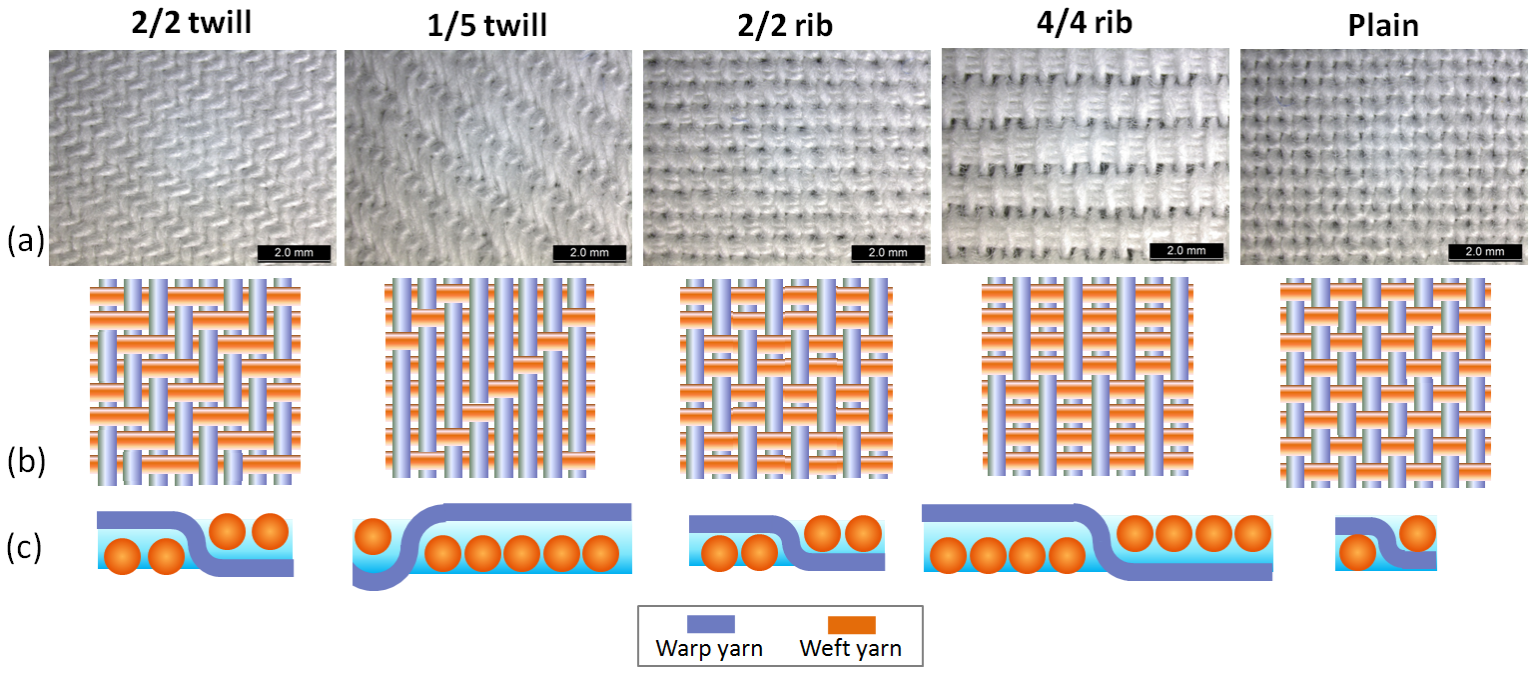
Image and illustration of Group A's fabrics. (a): Microscopic images of fabrics with different weave pattern. Here, the images of fabrics woven with 20/1 s cotton warp and 20/1 s cotton weft yarn were shown. It includes the images of fabric ‘2’, ‘6’, ‘12’, ‘16’ and ‘20’. Fabric ‘2’ represents 2/2 twill structure, fabric ‘6’ represents 1/5 twill structure, fabric ‘12’ represents 2/2 rib structure, fabric ‘16’ represents 4/4 rib structure and fabric ‘20’ represents plain structure. (b): Illustration of weave pattern with the warp yarn in purple colour and the weft yarn in orange colour. (c): Illustration of cross-section of fabrics showing the intersection of yarns.

Two-way ANOVA results (shown in Supplementary Table S4 online) suggest that the effect of structure is significant in the water absorption rate of fabric (p = 0.00 < 0.05). Figure 4(a) shows that the highest water absorption rate is found in fabrics made by 4/4 rib structure (i.e. fabric ‘16’ and ‘18’). Pairwise comparisons (shown in Supplementary Table S5) suggest that 4/4 rib structure are significantly higher than the other structures (p < 0.05). For the 4/4 rib fabrics, four weft yarns were grouped together intermittently, forming straight capillaries in the weft direction which are likely to promote liquid flow and liquid storage (as illustrated in Figure 5). This could explain the highest water absorption rate observed in this fabric structure. Figure 4(b) shows that the wetted area for the fabrics made by 45/2 s cotton yarn (i.e. fabric ‘4’, ‘8’, ‘14’, ‘18’, ‘22’) is generally larger than the one made by 20/1 s cotton yarn (i.e. fabric ‘2’, ‘6’, ‘12’, ‘16’, ‘20’). ANOVA results (presented in Supplementary Table S4 online) indicate that the effect of yarn is significant in the wetted area of fabric (p = 0.00 < 0.05). Greater number of smaller inter-yarn pores existed in fabrics woven by 45/2 s cotton yarn (i.e. two thinner yarns twisted together to form a weft yarn) and capillary theory shows that smaller pores result in higher capillary pressure and enhance liquid spreading distance^25^. This explains why larger wetted area is observed for fabrics made by 45/2 s cotton yarn. Pairwise comparisons demonstrate that the water content of 1/5 twill fabrics (i.e. fabric ‘6’ and ‘8’) is significantly lower than the other structures (as shown in Supplementary Table S5 online) and this could attribute to the higher porosity and thickness of this structure (as shown in Supplementary Table S3 online) which is likely to accommodate much more water.

For the 3-layer test, ANOVA test (as shown in Supplementary Table S4 online) shows that significant interaction effect of fabric structure and yarn occurs in the amount of water absorbed by bottom filter paper (p = 0.00 < 0.05) and transplanar ratio (p = 0.00 < 0.05). Turning this around, for example, a particular fabric structure is not always good at transplanar wicking and it depends on yarn type indeed and this can be observed in Figure 4(e).

Figure 4(d) shows that the amount of water left on the bottom filter paper is more when a plain fabric (i.e. fabric ‘20’ and ‘22’) was put on top of it, implying that plain fabric cannot pick up moisture as quick as the rest and transport to the outside environment efficiently. As illustrated in Figure 5, plain structure has more intersection points along the yarn and higher yarn tortuosity (i.e. more up and down along the yarn), resulting in difficulties in transporting water and this could account for the larger quantity of water absorbed in its bottom filter paper.

In summary, the aforementioned demonstrates that SUWTT is capable of differentiating fabrics with varying geometrical properties even same material was used to construct the fabric.

### Group B – Examining the sensitivity of the instrument

Due to the hydrophobic feature of fabric ‘2 W’, ‘3 W’, ‘5 W’, ‘10 W’, ‘20 W’ and ‘60 W’, water does not wick through the fabric under zero hydrostatic head, so the water absorption rate and wetted area are zero for these fabrics, implying poor absorbency. Without any water absorbed by the fabric in these samples, it is not applicable to calculate the water content of these fabrics as well.

Figure 4(a), Figure 4(b) and Figure 4(e) suggest that a low concentration of water repellent finish (0.5 ml/L of OLEOPHOBOL® CO finish) is favourable for cotton fabric in terms of water absorption rate, wetted area and transplanar ratio. The incorporation of water repellent finishing agent might decelerate the diffusion of water into interior fibre space of cotton and reduce fibre swelling^26^,^27^. To some extent, fibre swelling is an unfavourable feature for water transport since it narrows the gaps between the fibres^28^ and weakens the wicking effect. Compared with fabric ‘0.5 W’, fabric ‘WC’ (i.e. control fabric) with greater swelling, therefore, results in slower progression of liquid and worse performance in water transport. However, further increase in the concentration of water repellent finish may end up with poorer wicking properties. From Figure 4(d) and Figure 4(e), it can be observed that the amount of water absorbed by the bottom filter paper increases gradually while the transplanar ratio decreases sharply with higher concentration of water repellent finish, implying much water may leave on skin when sweating. These trends agree with our research hypothesis that water repellent finish is undesirable for water transport and can help to examine the sensitivity of this instrument.

### Group C – The flexibility in investigating high performance fabrics

The fabrics in this group were made either by specially designed structure or by specially engineered yarn. Fabric ‘PKnitPol’ was knitted by a specially designed plant structure whose advantages have been evaluated elsewhere^29^. The differential surface area between the inner and outer surface of the fabric and its branching network contribute to excellent moisture management property. On the other hand, fabric ‘Coolmax’ was knitted by a special yarn of which the channels on the yarn surface could facilitate wicking^30^,^31^.

Figure 4(a) demonstrates that the water absorption rate of these moisture management fabrics is higher than group A's and group B's fabrics. Figure 4(b) shows that their wetted area is larger whereas Figure 4(e) illustrates that the transplanar wicking property is better. The transplanar ratio for fabric ‘Coolmax’ is even larger than 1, implying that the amount of water transport away from skin is more than the amount stayed next to the skin layer. The larger spreading area and greater transplanar wicking could help to control the movement of body sweat in such a way that they are transported away from the skin to the outer surface of fabric where they can evaporate quickly. This rational finding suggests that this instrument is capable of characterising high performance moisture management fabrics.

## Conclusion

An instrument for measuring the in-plane and transplanar wicking properties of fabrics based on gravimetric and image analysis technique was developed. The wetting and wicking properties of textiles can be described comprehensively by a list of parameters investigated, including: (i) water absorption rate, (ii) wetted area, (iii) water content, (iv) amount of water absorbed by the bottom filter paper, and (v) transplanar ratio. These parameters were adopted to differentiate fabrics made by differing fabric structure and yarn, fabrics with different concentration of water repellent finish, and the moisture management fabrics. The uniqueness and advantages of the proposed instrument include:

- relate to wear comfort with the initial sweating period being investigated (how quick the sweat being absorbed),
- capable of measuring in-plane and transplanar wicking property of fabric in a simple way (relate to long-term wear comfort),
- direct and real-time monitoring of the water absorption amount,
- propose a novel parameter called ‘amount of water absorbed by the bottom filter paper’ and this reflects our skin condition when sweated,
- testing automation could even minimise variation caused by subjective manipulation, thus enhancing testing accuracy and reproducibility,
- versatile in terms of types of fabrics to be tested,
- continuous water supply simulating profuse sweating,
- the direction of water spread is close to the actual wear condition, and
- short testing duration.

With this instrument, fabrics can be characterized efficiently which helps to develop more functional and comfortable product. Compared with the conventional testing methods, SUWTT has improved in numerous aspects as shown in Supplementary Table S8.

## Author Contributions

K.P.M., Y.S. and K.H. constructed the instrument. K.H. calibrated the instrument. C.W. and J.T. gave scientific advice. K.P.M. interpreted data and wrote the manuscript. All authors contributed to discussion and reviewed the manuscript.

## Supplementary Material

Supplementary Information Supplementary Information

## References

[b1] Hollies N. R. S., Custer A. G., Morin C. J. & Howard M. E. A Human Perception Analysis Approach to Clothing Comfort. Text. Res. J. 49, 557–564 (1979).

[b2] Li Y., Plante A. M. & Holcombe B. V. The Physical Mechanisms of the Perception of Dampness in Fabrics. J. Therm. Biol. 18, 417–419 (1993). 10.2114/ahs1983.11.631 1476564

[b3] Fukazawa T. & Havenith G. Differences in comfort perception in relation to local and whole body skin wettedness. Eur. J. Appl. Physiol. 106, 15–24 (2009). 19159949 10.1007/s00421-009-0983-z

[b4] Galbraith R. L., Werden J. E., Fahnestock M. K. & Price B. Comfort of Subjects Clothed in Cotton, Water Repellent Cotton, and Orlon Suits. Text. Res. J. 32, 236–242 (1962).

[b5] Yokura H. & Sukigara S. Evaluation of the Wetness of Pantiliners. Text. Res. J. 80, 1643–1647 (2010).

[b6] Meng F., Ng S. F. F., Hui C. L. P., Li Y. & Hu J. An objective method to characterize moisture management properties of disposable diapers. Text. Res. J. 81, 1647–1654 (2011).

[b7] Elsner P., Hatch K. & Alberti W. W. Textiles and the Skin. Curr. Probl. Dermatol. 31, 24–34 (2003). 12882017 10.1159/000072235

[b8] Davies A. M. [Use of knitted spacer fabrics for hygiene applications.] Textiles for hygiene and infection control [Brian J. McCarthy, ed. (ed.)] [27–47] (Woodhead Publishing Limited, Oxford, 2011).

[b9] Berg R. W., Milligan M. C. & Sarbaugh F. C. Association of skin wetness and pH with diaper dermatitis. Pediatr. Dermatol. 11, 18–20 (1994). 8170842 10.1111/j.1525-1470.1994.tb00066.x

[b10] Kaatze U. & Hubner C. Electromagnetic techniques for moisture content determination of materials. Meas. Sci. Technol. 21, 1–26 (2010).

[b11] Karppinen T., Kassamakov I., Haeggstrom E. & Stor-Pellinen J. Measuring paper wetting processes with laser transmission. Meas. Sci. Technol. 15, 1223–1229 (2004).

[b12] Stor-Pellinen J., Haeggstrom E., Karppinen T. & Luukkala M. Air-coupled ultrasonic transmission measurement through paper during wetting. Meas. Sci. Technol. 13, 770–774 (2002).

[b13] Stämpfli R., Brühwiler P. A., Rechsteiner I., Meyer V. R. & Rossi R. M. X-ray tomographic investigation of water distribution in textiles under compression – Possibilities for data presentation. Measurement 46, 1212–1219 (2013).

[b14] Zhu C. & Takatera M. A new thermocouple technique for the precise measurement of in-plane capillary water flow within fabrics. Text. Res. J. 84, 513–526 (2013).

[b15] Raja D., Ramesh Babu V., Senthilkumar M., Ramakrishnan G. & Kannan N. A dynamic sweat transfer tester for analyzing transverse sweat transfer properties of multi-weave structure fabrics. J. Ind. Text. 44, 211–231 (2013).

[b16] Tang K. P. M., Kan C. W. & Fan J. T. Evaluation of water absorption and transport property of fabrics. Text. Prog. 46, 1–132 (2014).

[b17] AATCC 79 Absorbency of textiles. (American Association of Textile Chemists and Colorists, 2007).

[b18] AATCC 197 Vertical wicking of Textiles. (American Association of Textile Chemists and Colorists, 2011).

[b19] AATCC 195 Liquid Moisture Management Properties of Textile fabrics. (American Association of Textile Chemists and Colorists, 2009).

[b20] Morent R. *et al.* Measuring the wicking behavior of textiles by the combination of a horizontal wicking experiment and image processing. Rev. Sci. Instrum. 77, 093502 (2006).

[b21] Leisen J. *et al.* Through-plane diffusion of moisture in paper detected by magnetic resonance imaging. Ind. Eng. Chem. Res. 41, 6555–6565 (2002).

[b22] Howaldt M. & Yoganathan A. P. Laser-Doppler Anemometry to Study Fluid Transport in Fibrous Assemblies. Text. Res. J. 53, 544–551 (1983).

[b23] Lee J. H., Kim S. H., Lee K. J., Lim D. Y. & Jeon H. Y. Determining the absorption properties of split-type microfiber fabrics by measuring the change in color depth. Text. Res. J. 74, 271–278 (2004).

[b24] Miller B. & Tyomkin I. Spontaneous Transplanar Uptake of Liquids by Fabrics. Text. Res. J. 54, 706–712 (1984).

[b25] Hsieh Y.-L. Liquid Transport in Fabric Structures. Text. Res. J. 65, 299–307 (1995).

[b26] Kissa E. Capillary Sorption in Fibrous Assemblies. J. Colloid Interface Sci. 83, 265–272 (1981).

[b27] Kawase T., Sekoguchi S., Fujii T. & Minagawa M. Spreading of Liquids in Textile Assemblies. Part I: Capillary Spreading of Liquids. Text. Res. J. 56, 409–414 (1986).

[b28] Cil M. G., Nergis U. B. & Candan C. An Experimental Study of Some Comfort-related Properties of Cotton-Acrylic Knitted Fabrics. Text. Res. J. 79, 917–923 (2009).

[b29] Chen Q., Fan J. T., Sarkar M. & Jiang G. M. Biomimetics of Plant Structure in Knitted Fabrics to Improve the Liquid Water Transport Properties. Text. Res. J. 80, 568–576 (2010).

[b30] Zhang Y., Wang H. P. & Chen Y. H. Capillary effect of hydrophobic polyester fiber bundles with noncircular cross section. J. Appl. Polym. Sci. 102, 1405–1412 (2006).

[b31] Patnaik A., Rengasamy R. S., Kothari V. K. & Ghosh A. Wetting and Wicking in Fibrous Materials. Text. Prog. 38, 1–105 (2006).

