# Supplementary material for: Characterizing the transplanar and in-plane water transport of textiles with gravimetric and image analysis technique: Spontaneous Uptake Water Transport Tester
Source: Sci Rep. 2015 Apr 15;5:9689. doi: 10.1038/srep09689 (PMC4397916; doi:10.1038/srep09689)
Supplement: Supplementary Information [file srep09689-s1.pdf]

Supplementary Information For:

Characterizing the transplanar and in-plane water transport of textiles with gravimetric and image analysis technique: Spontaneous Uptake Water Transport Tester

K. P. M. Tang[1], Y. S. Wu[2], K. H. Chau[1], C. W. Kan[1*] and J. T. Fan[1,2**]
[1] Institute of Textiles and Clothing, The Hong Kong Polytechnic University, Hung Hom, Hong Kong
[2] College of Human Ecology, Cornell University, Ithaca, 14853, NY, United States

*Corresponding author: tccwk@polyu.edu.hk
**Corresponding author: jf456@cornell.edu

Introduction

Supplementary Table S1. Various conventional water absorption and transport test methods

Test method	Principle of the test	Advantages	Limitations/ Potential problems
Absorbency of textiles<br><br>(BS 4554 [1]; AATCC 79 [2])	The so-called 'Drop test' by observation to measure the absorption time of a droplet of liquid	- Simple<br>- Low cost<br>- Short operation time<br>- No specific equipment required	- Prone to subjective variations<br>- Discontinuous water supply<br>- Not applicable to highly absorptive fabric<br>- The use of sucrose solution (as specified in BS 4554) does not simulate the actual wear condition
Longitudinal wicking 'stripe' test<br><br>(AATCC 197 [3]; BE 3424-18 [4])	Record the initial and extended wicking rate of a vertically hanged fabric by observation	- Simple<br>- No specific instrument required	- Difficult in observing the liquid front line<br>- The direction of water spread have limited implication in clothing comfort<br>- Not accessible to study the moisture distribution within a textile layer<br>- Not suitable for dark-coloured fabrics<br>- Time consuming for fabric preparation
Horizontal wicking test<br><br>(AATCC198 [5])	Record the horizontal wicking rate of fabric by observation	- Simple<br>- No specific instrument required<br>- Short testing duration	- The volume of material being wet might not be proportional to the wetted area → Poor accuracy<br>- Not suitable for dark-coloured fabric or fabrics with poor absorbency
Moisture management Tester<br><br>(AATCC 195 [6])	Measure the change in electrical resistance in different portions and sides of the fabric	- Measurement is done automatically which is more reliable<br>- User-friendly<br>- Applicable to dark-coloured fabrics and fabrics with high absorption rate	- The drop of water was from top of fabric of which the gravity-induced pressure may vary the result<br>- Does not resemble the profuse sweating condition<br>- Not suitable for long pile fabrics, conductive material, coated, laminated fabrics or complex fabric constructions
Gravimetric Absorption Testing System (GATS)<br><br>(McConnell, 1982 [7]; Yoo and Barker, 2004 [8])	Gravimetric method by recording the change in water loss of the water reservoir	- Profuse sweating condition is closer to actual wear condition<br>- Averaging the wicking in lengthwise and widthwise directions<br>→ Eliminate the number of test	- The repeatability of the instrument depends on the accuracy of the shaft<br>- Not capable of differentiating wicking from in-plane and transplanar direction
Transplanar Water Transport Tester (TWTT)<br><br>(Sarkar et al., 2007 [9])		- Profuse sweating condition<br>- Constant water level<br>→ accurate<br>- The sample was not compressed<br>→ reflecting the actual absorption performance<br>- Automatic sample positioning<br>→ reproducible	- The fabric is prone to have uneven contact with the sample podium<br>- Not capable of differentiating wicking from in-plane and transplanar direction

Supplementary Table S2. Summary of the advantages and disadvantages of each technique

<table>
<tr><th>Technique</th><th>Advantages</th><th>Disadvantage</th><th>Specific Equipment required</th></tr>
<tr><td>Gravimetric</td><td>- Simple
- Possible to record the time-dependence curve at regular time intervals in real-time
- Applicable to fabrics with irregular spreading behaviour
- Dye solution is not necessary to trace the liquid front
- Versatile (Not affected by the physical appearance of the material to be tested)
- Reduce manual manipulation which improve testing accuracy</td><td>- Inability to study the space-dependence of moisture distribution within a textile layer
- The first few seconds of the measurement are strongly affected by the way to place the specimen onto the balance or the way to deliver water to the fabric</td><td>Balance</td></tr>
<tr><td rowspan="2">Observation-based</td><td rowspan="2">- Low cost
- No specific equipment required</td><td>- Prone to be interfered by subjective factors
- Not applicable to test highly absorptive fabrics in a repeatable manner</td><td rowspan="2">nil</td></tr>
<tr><td rowspan="2">- The use of dye solution to amplify the wicking effect may change the surface tension of the testing liquid, limiting its value in investigating clothing comfort
- Inconsistent lighting of the testing environment may affect the detection of the wetted pattern
- Not suitable for dark-coloured fabrics or samples with differential wettability in their surfaces
- The uneven spreading pattern may complicate the measurement
- The spreading area is not always related to the volume of the material being wetted</td></tr>
<tr><td>Optical</td><td>- Possible to record the time-dependence curve at regular time intervals in real-time
- Reduce manual manipulation which improve testing accuracy</td><td>Camera, video recorder</td></tr>
<tr><td>Spectroscopic</td><td>- Precise and comprehensive method
- Capable of studying the dynamics of moisture transport without disturbing a textile assembly during measurement
- Quantitatively monitor the internal moisture distribution of a textile layer
- Suitable for fabrics with asymmetrical capillary transport and multilayer material
- Not affected by optical opaqueness of the material</td><td>- Specific and very expensive equipment is needed
- Complicated operation
- The risk of X-ray to human health
- The use of contrast agent (µCT) or $CuSO_4$ solution (NMR) may form residue on the fabric when water evaporate from the fabric which affect testing accuracy</td><td>Spectrophotometer, X-ray microtomography, Nuclear magnetic resonance spectroscopy and neutron radiography system</td></tr>
<tr><td rowspan="2">Electrical</td><td>- Indirect prediction of fabric’s water content</td><td rowspan="2">- Not suitable for testing conductive material
- The use of sodium chloride solution may oxidize the electronic parts of the instrument which affect testing accuracy
- The result may vary with the fabric roughness (contact between the electrical sensor and fabric)</td><td rowspan="2">High-resistance mega ohmmeter, and electrical sensor such as multiple capacitance transducers</td></tr>
<tr><td rowspan="4">- Possible to record the time-dependence curve at regular time intervals in real-time
- Applicable to fabrics with irregular spreading behaviour
- Dye solution is not necessary to trace the liquid front
- Versatile (Not affected by the physical appearance of the material to be tested)
- Reduce manual manipulation which improve testing accuracy</td></tr>
<tr><td>Pressure-based</td><td>- Not versatile (can be applied to limited types of fabrics)</td><td>Pressure transducer</td></tr>
<tr><td>Magnetic resonance</td><td>- Complicated set-up</td><td>Inductive sensor</td></tr>
<tr><td>Temperature detection</td><td>- The result may vary with the fabric roughness (contact between the temperature sensor and fabric)
- The ambient condition should be properly controlled, otherwise, it will affect testing accuracy</td><td>Temperature sensor</td></tr>
</table>

Methods

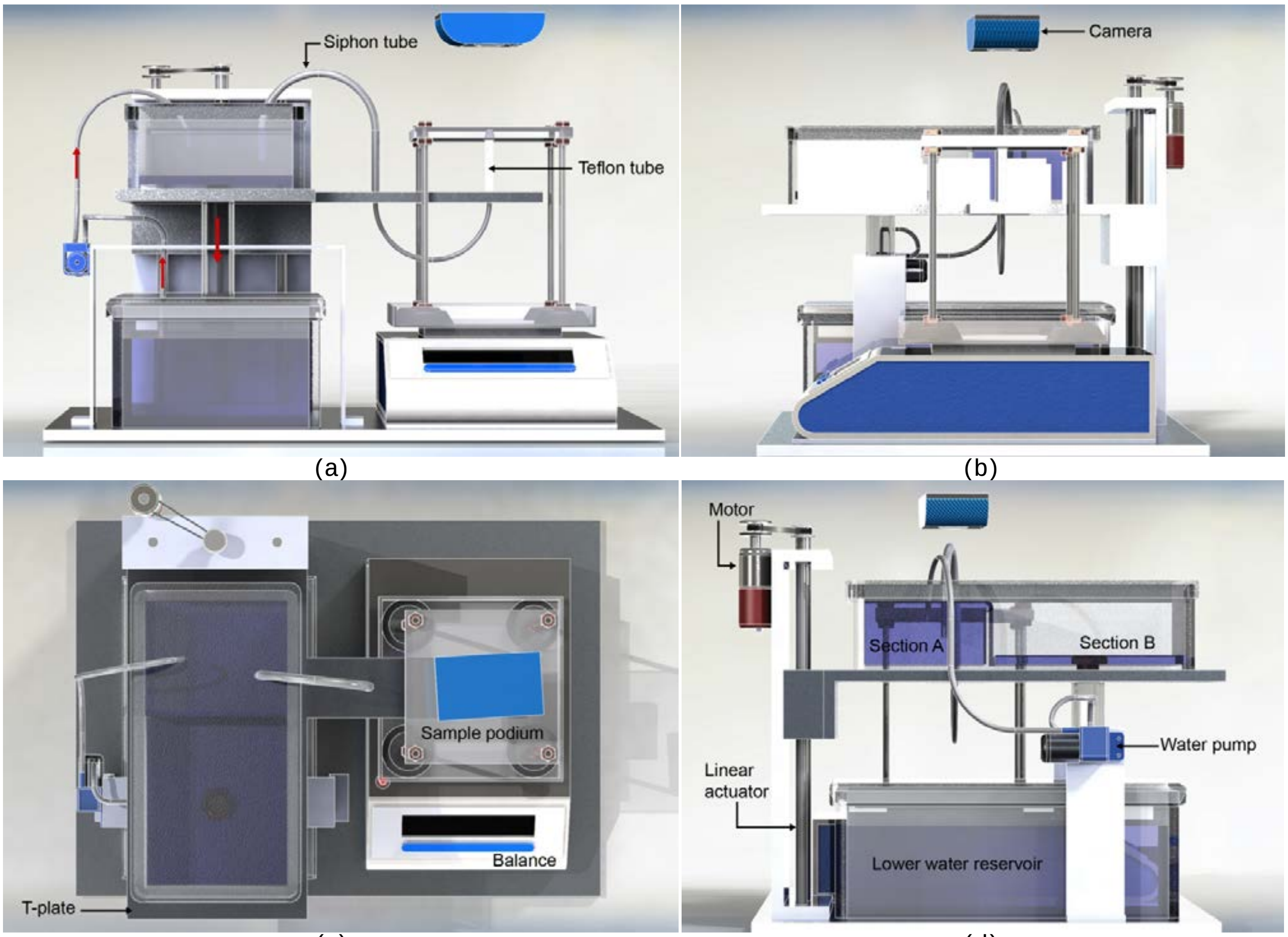

(a) (b)

(c) (d)

Supplementary Figure S1. SUWTT from different viewpoints. (a) Front view, (b) Right view, (c) Top view, and (d) Left view

Components selection and specification

In order to minimise the amount of liquid that might transport within the fabric-plate interface, a non-wettable surface is preferred and a polytetrafluorethylene (PTFE) plate was selected. To enable automatic movement of the T-plate, a robust motor (980D28121, MFA) was used and its movement speed varies with the supply voltage. The T-plate was raised at the speed of 2.7 mm/min (with the use of - 3.5 V) while it was lowered at 6.9 mm/min (with the application of + 7.5 V). To ensure steady water level of the upper water tank, a micropump (D250-03, RS) with stable and relatively low flow rate (40 ml/min at maximum voltage supply) was chosen. To facilitate real-time monitoring of the water absorption amount in fabric, a balance (MS1003S, Mettler Toledo), with 0.001 g repeatability and maximum response rate of 22.9 data per second, was utilised. It is capable of connecting to the PC with RS-232C. To connect various electronic components with the computer for testing automation, data acquisition device (DAQ 6525, National Instruments) was used. It features digital input function for sensing DC voltage and digital output function for controlling the external voltage applied to motor for moving the T-plate. Two photoelectric sensors (EE-SX 671, ORMON) were wired to the digital input channels of the DAQ device. When the opaque plate reached the slot of the photoelectric sensor, a signal was generated and transferred to the computer via DAQ device. The feedback signal was then returned to DAQ device to disconnect voltage supply to motor. Therefore, the Teflon tube mounted on T-plate, with the aid of photoelectric sensor, was brought to the same height level for supplying water to the fabric in a repeatable manner.

Control programming

LabVIEW 2009 programming was used to develop an interface for controlling the movement of the T-plate, receiving input signal, performing logical decision, recording water uptake rate and analysing data. Figure S2 shows the control interface for SUWTT and it is divided into four parts as denoted by the number indicated in the figure. Part 1 is for inputting sample details, setting the testing duration, and controlling the start and end of the test. Part 2 is for recording the weight of the fabric before and after the test, and the mass of water absorbed by each layer can be calculated automatically. Part 3 is to capture the wetted pattern and by calculating the number of pixels in the recorded image, the wetted area was then known. Part 4 shows the time-dependence absorption curve of the sample in real-time.

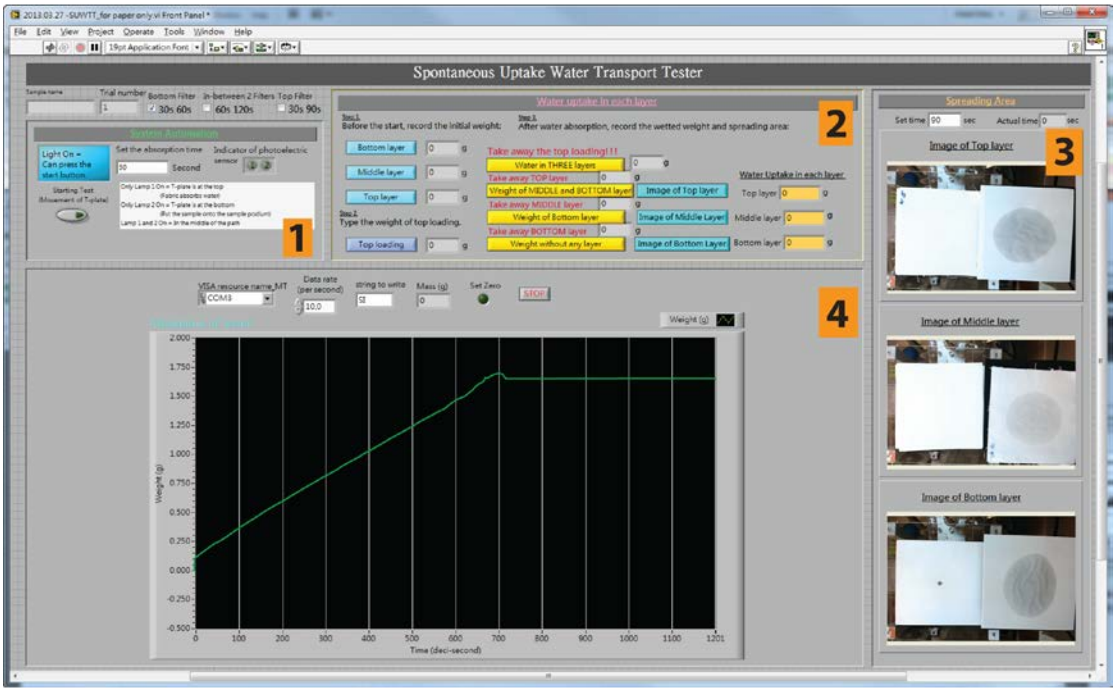

Supplementary Figure S2. SUWTT Control interface

Experimental details – Sample

The porosity of the fabric is calculated according to equation (1) with reference to Hsieh's work [10].

$$\text{Porosity} = 1 - \frac{\text{Fabric weight } (\text{g}/\text{cm}^2)/\text{ Fabric thickness (cm)}}{\text{Bulk density of fibre } (\text{g}/\text{cm}^3)} \qquad (1)$$

Supplementary Table S3. Details and specifications of various fabrics

	Fabric details	Fabric code.	Concentration of OLEOPHOBOL® CO finishing (ml/L) ◇	Construction		Fibre content	Fabric density		Weight (g/m^2)	Thickness (mm)	Porosity	Water absorption time (s)‡ ¶
				Type	Structure		epi	ppi				
Group A	Thick shirting fabrics with different fabric structure and yarn type which are made by the same material using a 16 heald dobby loom	2 §	0	Woven	2/2 twill	Cotton	77.4	94.6	207.36	0.596	0.7741	0.8 (18.4)
		4 †	0				77.4	96.8	196.26	0.580	0.7803	0.6 (15.2)
		6 §	0		1/5 twill		78.4	89.4	220.52	0.828	0.8271	0.3 (14.7)
		8 †	0				80.2	89.2	201.08	0.800	0.8368	0.4 (16.8)
		12 §	0		2/2 rib		73.8	101.8	210.14	0.508	0.7314	1.1 (14.0)
		14 †	0				71.8	100.8	193.62	0.488	0.7424	1.1 (15.3)
		16 §	0		4/4 rib		69.2	105.6	204.08	0.724	0.8170	0.4 (7.1)
		18 †	0				68.2	106.2	191.34	0.712	0.8255	0.3 (25.7)
		20 §	0		Plain		73.4	73.4	179.28	0.472	0.7534	1.5 (7.6)
		22 †	0				76.2	73.8	163.54	0.432	0.7542	1.2 (7.0)
Group B	Plain cotton fabrics finished with different degrees of OLEOPHOBOL® CO finishing (number in the fabric code indicating the concentration of finish in ml/L)	WC	0				140	75	121.95	0.288	0.7250	2.8 (21.6)
		0.5W	0.5				142	75	124.60	0.336	0.7592	1.2 (15.2)
		1W	1				141	75	124.00	0.340	0.7632	1.7 (23.5)
		2W*	2				140	74	124.95	0.340	0.7614	60+ (0)
		3W*	3				142	75	124.70	0.344	0.7646	60+ (0)
		5W*	5				142	74	124.40	0.356	0.7731	60+ (0)
		10W*	10				142	75	124.90	0.360	0.7747	60+ (0)
		20W*	20				141	74	124.50	0.356	0.7729	60+ (0)
		60W*	60				140	74	125.20	0.352	0.7690	60+ (0)
Group C	Moisture management fabric by specially designed fabric structure or the use of specially engineered yarn	PKnitPol	0	Knitted	Double jersey plant structure	Polyester	51	59	117.00	0.520	0.8381	0.8 (10.0)
		Coolmax	0		Plain knit	Polyester (Coolmax™ Δ)	35	70	129.58	0.512	0.8179	0.3 (8.8)

◇ OLEOPHOBOL® CO finishing is a kind of water repellent finishing agent by HUNTSMAN and was applied to the fabric through pad-dry-cure process.

‡ Measured according to AATCC 79 - Absorbency of textiles with 12 replicates

¶ With CV % shown in parentheses ( )

§ Fabrics made by 20/1 s cotton weft yarn

† Fabrics made by 45/2 s cotton weft yarn

* Hydrophobic samples (as measured by AATCC 79 with water absorption time longer than 60 seconds)

Δ Coolmax® fibres are oblong in cross-section with grooves running lengthwise along the threads. The closely spaced channels facilitate wicking of water and as a result increasing the evaporation rate.

Operational Testing Procedures

Prior to the test, the instrument was ‘warmed up’ for 30 minutes and the ordinary testing procedure is listed below:

- Set the water supply duration in the LabVIEW control interface (Part 1 of Supplementary Figure S2).
- Check the water level in the bottom water reservoir and pour water to Section A of the upper water tank, if necessary.
- Place the first specimen onto the sample podium and record its dry weight in the LabVIEW control interface (shown in Part 2 of Supplementary Figure S2) and repeat the same procedure for the remaining two layers if 3-layer test is to be conducted.
- Apply an external pressure of 360 g (2.5 g/cm^2) onto the sample and tare the balance.
- Press the ‘start and record’ button in SUWTT control interface (shown in Part 1 of Supplementary Figure S2).
    - The program starts logging data.
    - The T-plate starts moving up until the water from the orifice of Teflon tube approaches the sample.
- After pre-determined duration, the T-plate is brought to the original position.
- Take away the external weight and record the amount of water uptake by each layer (shown in Part 2 of Supplementary Figure S2) and capture its spreading pattern correspondingly (shown in Part 3 of Supplementary Figure S2), starting from the top filter paper to the fabric layer and the bottom filter paper.

Results and Discussion

Supplementary Table S4. Two-way ANOVA test result for Group A's fabric

		1-layer test			3-layer test	
		Water absorption rate	Wetted area of fabric	Water content of fabric	Amount of water absorbed by bottom filter paper	Transplanar ratio
Sig.	Fabric Structure	0.000	0.000	0.000	0.000	0.012
	Yarn	0.926	0.000	0.000	0.042	0.000
	Fabric Structure*Yarn	0.167	0.496	0.041	0.002	0.000
R squared		0.830	0.867	0.975	0.895	0.898

Supplementary Table S5. Pairwise comparisons of various fabric structure on a list of water absorption and transport properties

Fabric structure		Water absorption rate		Wetted area of fabric		Water content of fabric		Amount of water absorbed by the bottom filter paper		Transplanar ratio	
(I)	(J)	Mean Difference (I-J)	Sig.	Mean Difference (I-J)	Sig.	Mean Difference (I-J)	Sig.	Mean Difference (I-J)	Sig.	Mean Difference (I-J)	Sig.
2/2 twill	1/5 twill	-.00491	.000	-9.552	.000	.106	.000	.046	.002	-.055	.005
	2/2 rib	.00010	.892	-6.309	.001	-.052	.000	-.039	.005	-.042	.044
	4/4 rib	-.00646	.000	-16.179	.000	.074	.000	-.008	.520	-.068	.002
	plain	-.00052	.485	-17.259	.000	-.051	.000	-.122	.000	-.028	.115
1/5 twill	2/2 twill	.00491	.000	9.552	.000	-.106	.000	-.046	.002	.055	.005
	2/2 rib	.00502	.000	3.243	.062	-.157	.000	-.085	.000	.013	.506
	4/4 rib	-.00154	.037	-6.627	.000	-.032	.000	-.054	.000	-.012	.521
	plain	.00439	.000	-7.707	.000	-.157	.000	-.168	.000	.027	.147
2/2 rib	2/2 twill	-.00010	.892	6.309	.001	.052	.000	.039	.005	.042	.044
	1/5 twill	-.00502	.000	-3.243	.062	.157	.000	.085	.000	-.013	.506
	4/4 rib	-.00656	.000	-9.870	.000	.125	.000	.031	.027	-.026	.231
	plain	-.00062	.371	-10.950	.000	.000	.938	-.083	.000	.013	.507
4/4 rib	2/2 twill	.00646	.000	16.179	.000	-.074	.000	.008	.520	.068	.002
	1/5 twill	.00154	.037	6.627	.000	.032	.000	.054	.000	.012	.521
	2/2 rib	.00656	.000	9.870	.000	-.125	.000	-.031	.027	.026	.231
	plain	.00594	.000	-1.080	.499	-.125	.000	-.114	.000	.039	.052
plain	2/2 twill	.00052	.485	17.259	.000	.051	.000	.122	.000	.028	.115
	1/5 twill	-.00439	.000	7.707	.000	.157	.000	.168	.000	-.027	.147
	2/2 rib	.00062	.371	10.950	.000	.000	.938	.083	.000	-.013	.507
	4/4 rib	-.00594	.000	1.080	.499	.125	.000	.114	.000	-.039	.052

Supplementary Table S6. Pairwise comparison of different yarn type on various water absorption and transport properties

Yarn type		Water absorption rate		Wetted area of fabric		Water content of fabric		Amount of water absorbed by the bottom filter paper		Transplanar ratio	
(I)	(J)	Mean Difference (I-J)	Sig.	Mean Difference (I-J)	Sig.	Mean Difference (I-J)	Sig.	Mean Difference (I-J)	Sig.	Mean Difference (I-J)	Sig.
20/1s cotton	45/2s cotton	-4.276E-5	.926	-6.781	.000	.020	.000	-.018	.042	-.015	.000

Precision of the equipment

As the purposed instrument is based on gravimetric technique, the precision of the electronic balance (Mettler Toledo MS1003S), a dominant component for characterising fabric's absorbency in this study, should be reported and is found to be ± 0.001g. Also, image analysis technique is adopted for characterising the wetted area. To ensure accurate measurement, the camera was mounted to a fixed position attached with constant lighting.

Uncertainty of different parameters

Some parameters such as water content and transplanar ratio cannot be measured in a single measurement. In fact, several physical factors are involved and the uncertainty of these parameters can be calculated from the uncertainty of each direct measurement [11]. The calculation of uncertainty of water content is based on the assumption that all errors are independent and random, and it is computed by the quadratic sum according to equation (2). For the transplanar ratio, the water absorption amount by top filter paper is divided by and interrelates with the absorption amount in the bottom filter paper, so the uncertainty of transplanar ratio is calculated by the sum of fractional uncertainties in two direct measurements, as shown in equation (3).

$$\text{Uncertainty of Water content} = \sqrt{\left(\frac{\delta_{\text{Mass of water absorbed}}}{\text{Mass of water absorbed}}\right)^2 + \left(\frac{\delta_{\text{Wetted area}}}{\text{Wetted area}}\right)^2 + \left(\frac{\delta_{\text{Thickness}}}{\text{Thickness}}\right)^2 + \left(\frac{\delta_{\text{Porosity}}}{\text{Porosity}}\right)^2} \quad (2)$$

$$\text{Uncertainty of Transplanar ratio} = \frac{\delta_{\text{Water absorbed by Filter paper Top}}}{\text{Water absorbed by Filter paper}_{\text{Top}}} + \frac{\delta_{\text{Water absorbed by Filter paper Bottom}}}{\text{Water absorbed by Filter paper}_{\text{Bottom}}} \quad (3)$$

where δ is the measured uncertainty from each component.

For the fabrics investigated in the presented study, the average uncertainty of water content of fabric is $(\sqrt{6.243^2 + 3.632^2 + 2.655^2 + 0.630^2})$ % = 7.721 % while the average uncertainty for transplanar ratio is (5.250 + 4.786) % = 10.036 %.

Reproducibility of the SUWTT

In order to ensure the result is reproducible and reliable, it is found necessary to have a routine calibration of the entire set up. This may be done by testing a standard material for a pre-determined time and measuring for its weight and spreading area. A conventional filter paper with guarantee wetting performance was tested and the coefficient of variation (CV) of the collected amount and the spreading area for this standardised material is within 4 % for five replicates, it can be concluded that the setting is well calibrated.

To investigate the reproducibility of this test, the CV % of various measurement parameters is examined. For a 1-layer test, the average CV % of water absorption rate and wetted area for 15 types of fabrics is 6.086 and 3.632, respectively. For a 3-layer test, the average CV % of amount of water absorbed by the bottom filter paper for 21 types of fabrics is 4.786. The results indicate that the reproducibility of the test is quite high. Comparatively, the CV % of the samples investigated is higher than that for the standardised material. It can be inferred that the fabric itself might contribute to the variance of the result since these samples are produced and finished in laboratory scale equipment, but not a commercialised set up. The CV % of AATCC Test method 79 - Absorbency of textiles also demonstrates the variability of these samples, suggesting the error of the result might come from the fabric itself.

Accuracy of SUWTT

In order to check the accuracy of the proposed set up, the SUWTT result was correlated with the one measured by the conventional measurement methods, such as wettability test (AATCC 79) [2], vertical wicking test (AATCC 197) [3], horizontal wicking test [12], moisture management tester (AATCC 195) [6] and water absorption capacity test [12]. For easy and better understanding about these methods, the testing principles are summarised in Supplementary Table S7.

Supplementary Table S7. Testing principles of various objective measurements

Test methods	Testing principles	Measurement parameters
Wettability test (AATCC 79)	A drop of water was delivered from a fixed height onto the test sample. The time it takes for the drop of water to disappear was taken as a measure of the wettability of fabric. The shorter the time, the much more wettable the fabric is.	- Water absorption time (s)
Vertical wicking test (AATCC 197)	A preconditioned strip of the specimen was suspended vertically with its lower end immersed in a reservoir of distilled water and the height of water reached in the fabric against gravity was visually observed and recorded after a fixed time. The initial and extended wicking rate, expressed in mm/s, indicates the average speed of water to reach 20 and 150 mm height, respectively.	- Initial wicking rate, 20 mm divided by time spent (mm/s)<br>- Extended wicking rate, 150 mm divided by time spent (mm/s)
Horizontal wicking test (Tang et al., 2014)	A fixed amount of water was supplied at the bottom side of fabric at a constant rate (10 ml/h). A camera, standing on top of the set-up, was utilized to capture the image of the wetted sample and the water spreading area was measured.	- Horizontal wicking area (cm^2)
Moisture management tester (AATCC 195)	The sample was put in-between two sets of metal electrodes and a fixed quantity of liquid was dropped onto the back side of the fabric and the direction of water spread was traced automatically by the metal electrodes.	- Overall (liquid) moisture management capability (OMMC)
Water absorption capacity test (Tang et al., 2014)	Fabric was put into a tank of water and 5 minutes was allowed for it to sink completely into the water. The fabric was then taken out by tweezers and hung onto a rod vertically until there was no water dripping within a 30-second interval. The water gain in fabric was measured and it is expressed as mass of water gain per unit gram of fabric in percentage.	- Wet pick-up (%)

The correlation results of various testing methods against SUWTT are illustrated in Figure S3 to Figure S6. Figure S3 shows that water absorption rate by SUWTT has moderate correlation with water absorption time by wettability test (Adj. R^2=0.68), initial wicking rate by vertical wicking test (Adj. R^2=0.69) and extended wicking rate by vertical wicking test (Adj. R^2=0.69). Higher absorption rate under spontaneous uptake (i.e. by SUWTT) is associated with shorter water absorption time and higher vertical wicking rate. This direction of correlation is rational and the strength of correlation is acceptable, thus it suggests that water absorption rate is measured accurately.

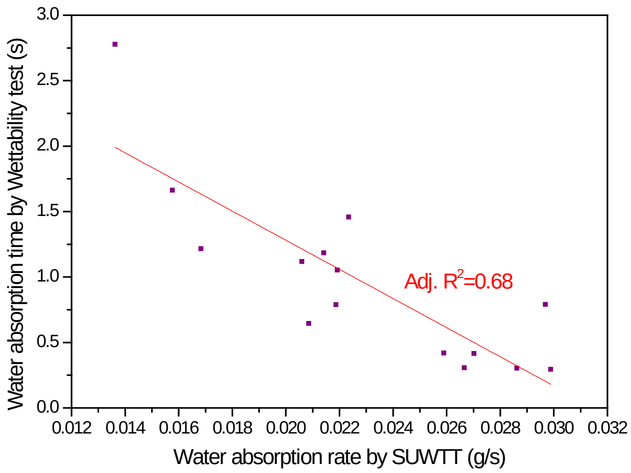

(a)

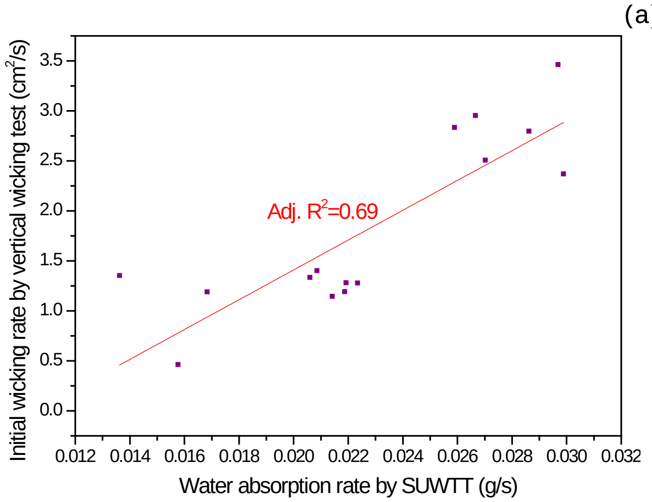

(b)

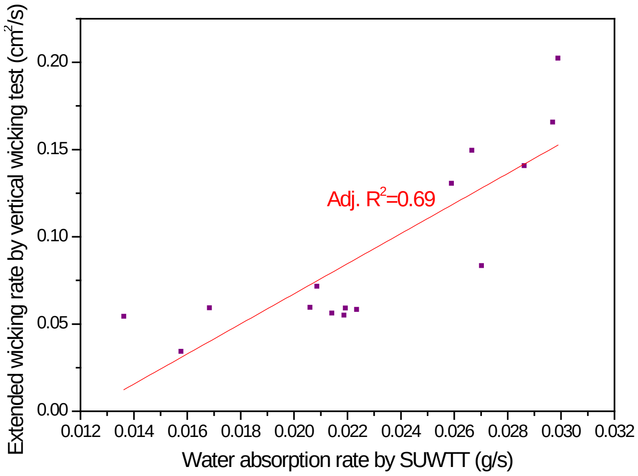

(c)

Supplementary Figure S3. Correlation of water absorption rate by SUWTT against (a) water absorption time by wettability test, (b) initial wicking rate by vertical wicking test, and (c) extended wicking rate by vertical wicking test

For the 1-layer SUWTT test, the absorption amount of the sample was recorded after 30 second water absorption. It is somehow related to the absorption capacity of the fabric and its correlation with the one by Tang et al.'s method [12] was studied. Figure S4 illustrates that it has moderate and positive correlation with wet pick-up (Adj. R^2=0.59) and this finding is understandable, implying the accuracy of this measurement.

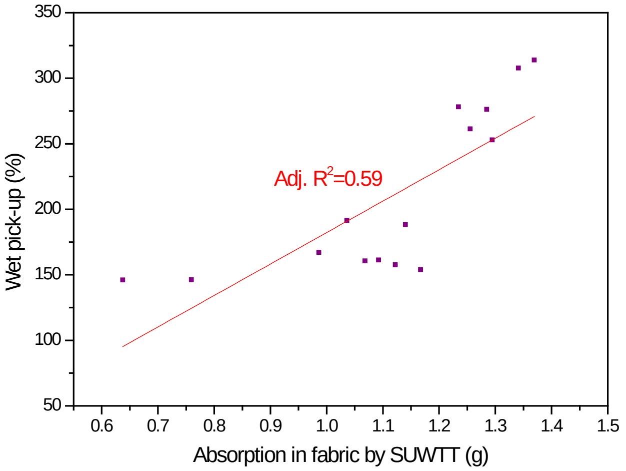

Supplementary Figure S4. Absorption in fabric by SUWTT against wet pick-up (%)by Water absorption capacity test

For the 1-layer SUWTT test, the wetted area of the sample was measured under demand wetting principle (i.e. the amount of water supply varies). This factor is affected by the wicking property and absorption capacity of the fabric. In order to standardise the measurement, the wetted area was divided by absorption amount of the sample and this was correlated with horizontal wicking area by Tang et al.'s [12] method. Figure S5 illustrates the correlation of wetted area by the two methods and these two factors are moderately correlated (Adj. R^2=0.67).

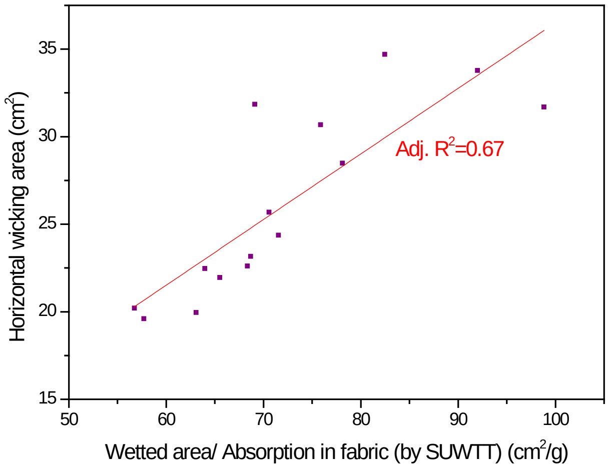

Supplementary Figure S5. Wetted area per unit gram of water by SUWTT against horizontal wicking area

For the 3-layer SUWTT test, the transplanar ratio which reflects the transplanar wicking property of fabric was measured. Figure S6 demonstrates that it is strongly related to OMMC by MMT (Adj. R^2=0.80). OMMC is an indirect measurement of moisture management property of fabric which is done by electrical method. A strong correlation implies that transplanar ratio by SUWTT gives accurate result.

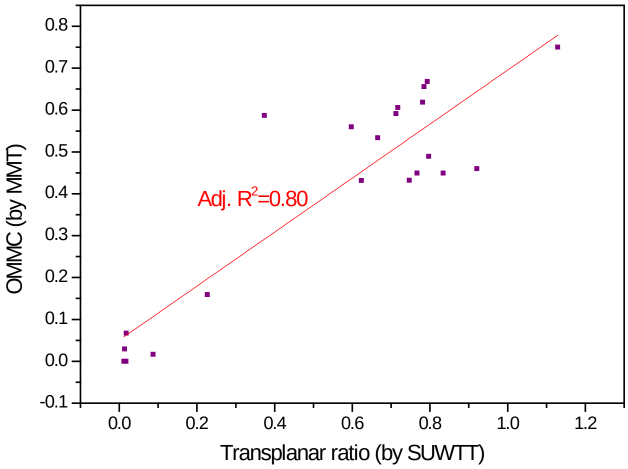

Supplementary Figure S6. Transplanar ratio by SUWTT against OMMC by MMT

On the other hand, for the conventional testing methods, 'the water content of fabric' and 'the amount of water absorbed by the bottom filter paper' cannot be measured and so the accuracy of these two measurements cannot be verified. In brief, SUWTT can perform accurate measurement with comprehensive information provided under short testing time.

Conclusion

Supplementary Table S8. Comparisons with the conventional testing methods

Test method	Limitations	Is the problem solved by SUWTT?
Drop test (AATCC 79)	Does not simulate the profuse sweating condition	✓
	The determination of end-point of testing is prone to subjective variation	✓
Vertical wicking test (AATCC 197)	The direction of water transport does not simulate actual use	✓
	The difficulty in tracing the position of liquid front by observation	Camera is used to capture the digital image of the wetted pattern and specific software was used to detect the liquid front. It may be difficult for some particular fabrics (e.g. dark-coloured fabric), but the calculation of wetted area will be more precise.
Horizontal wicking test (AATCC 198)		
	Water is applied from the top of fabric and gravity induced pressure may affect the wicking property	✓
Moisture Management Tester (AATCC 195)		
	Not suitable for fabrics with rough surface and thin fabrics	✓
	Not suitable for hydrophobic fabrics	Hydrophobic fabrics are only applicable to the 3-layer SUWTT test which means only the transplanar wicking property could be measured, but not for the water absorption rate.
Contact angle measurement	Not applicable to testing material which absorbs water very fast	✓
Gravimetric Absorption Testing System (GATS)	Cannot differentiate the direction of water flow within the fabric	✓
Transplanar Water Transport Tester (TWTT)		
	A slight deviation on the evenness of surface might affect the contact with the plate, thus affecting the accuracy of result	✓
	Only one layer of fabric could be tested	✓
Water absorption capacity measurement	Fabric saturation is considered, but the initial wetting performance is not examined.	✓
Spectroscopic method (e.g. MRI and NMR)	Expensive equipment and complicated handling	✓

Reference

1 *BSI 4554 Method of Test for Wettability of Textile fabrics*. (British Standards Institution, 1970).

2 *AATCC 79 Absorbency of textiles*. (American Association of Textile Chemists and Colorists, 2007).

3 *AATCC 197 Vertical wicking of Textiles*. (American Association of Textile Chemists and Colorists, 2011).

4 *BS EN 3424-18 Testing coated fabrics - Part 18: Methods 21A: Methods for determination of resistance of resistance to wicking*. (British Standards Instituion, 1986).

5 *AATCC 198 Horizontal wicking of Textiles*. (American Association of Textile Chemists and Colorists, 2011).

6 *AATCC 195 Liquid Moisture Management Properties of Textile fabrics*. (American Association of Textile Chemists and Colorists, 2009).

7 McConnell, W. J. Gravimetric Absorbency Tester. Wallingford, USA patent (1982).

8 Yoo, S. & Barker, R. L. Moisture management properties of heat-resistant workwear fabrics - Effects of hydrophilic finishes and hygroscopic fiber blends. *Text. Res. J.* **74**, 995-1000, (2004).

9 Sarkar, M., Fan, J. & Qian, X. Transplanar Water Transport Tester for Fabrics. *Meas. Sci. Technol.* **18**, 1465-1471, (2007).

10 Hsieh, Y.-L. Liquid Transport in Fabric Structures. *Text. Res. J.* **65**, 299-307, (1995).

11 Taylor, J. R. [An Introduction to Error Analysis : The Study of Uncertainties in Physical Measurements] [45-92] (University Science Books, United States, 1997).
12 Tang, K. P. M., Kan, C. W. & Fan, J. T. Assessing and Predicting the Subjective Wetness Sensation of Textiles: Subjective and Objective Evaluation. *Text. Res. J.*, (2014).
